# Alteration of Membrane Permeability of Bacteria and Yeast by High Frequency Alternating Current (HFAC)

**DOI:** 10.2174/1874285800802010032

**Published:** 2008-04-15

**Authors:** C Cassanelli, A Marchese, S Cagnacci, E.A Debbia

**Affiliations:** Sez. Microbiology- DISCAT, University of Genova, Largo Rosanna Benzi 10, 16132 Genova, Italy

**Keywords:** Plasmid transfer, membrane permeability, high frequency alternating current, re-growth suppression

## Abstract

**Aims:**

Endox^®^ Endodontic System (Endox) is used for endodontic treatment by a high frequency alternating current (HFAC). This device damaged the envelopes of spores and vegetative organisms. If the integrity of the envelope is compromised, the transit of compounds in the two directions is possible. This latter aspect was investigated here.

**Methods:**

The instrument delivered a 60ms pulse at a frequency 300 kHz, and power 800 KV/m. DNA transfer was verified using *Escherichia coli* K-12 strain carrying a non conjugative plasmid pBP517 (*gyr*A^+^) as donor and a rifampicin and nalidixic acid resistant recipient. 0.2 ml of mixture of donor and recipient strains in saline was exposed to HFAC and plated on selective media. Uptake of antimicrobials and a delay in re-growth was assessed exposing the strains to HFAC.

**Results:**

Plasmid transfer was detected under different experimental conditions. From 9 to 27 recombinants were found. Representative recombinants cured from plasmid showed the original phenotype. HFAC promoted the uptake of ineffective antibiotics, and induces a 1 h of delay in re-growth on the strains.

**Conclusions:**

Endox exhibited an effect on microrganisms which is reminiscent with that occuring in electroporation, but with a mode of action that saved materials and time.

## INTRODUCTION

Endox is an innovative method for the treatment of bacterial infections of the root canal [1]. This equipment uses a fine surgical steel needle as an active electrode which is introduced into the open root canal and a neutral electrode that is held in the patient's hand. Then a discharge of high frequency alternating current (HFAC) is applied. The passage of this electromagnetic field, when carried out under specific conditions of impedance, produces a sterilization of the root canal similar to that observed with standard methods using chemicals, with beneficial effects for inside and outside the tooth [1-2].

Using an experimental model simulating an *in vivo* situation, in fact, Endox demonstrated a lethal activity on different microorganisms representative of gram-positive and gram-negative bacterial species as well as *Candida albicans, Actinomyces *spp, and *Bacillus subtilis* spores [3].

Although the mode by which this instrument causes the death of the bacteria is not known, the damages generated by this high frequency alternating current are reminiscent with that observed with traditional electroporation methods [4]. Therefore, it has been assumed, that Endox kills bacteria by inducing pore formation and other defects in the envelope of the exposed organisms. If this hypothesis is correct, bacteria treated with HFAC generated by this apparatus could acquire genetic material or any sort of compound from the environment. Therefore in this study the transfer of a non conjugative plasmid from a donor to a recipient *Escherichia coli* strain exposed together to the high frequency alternating current generated by Endox was assessed. The uptake of various antibiotics in different organisms was also evaluated.

It is known that when micro-organisms are exposed to chemical or physical agents for a short period of time, a delay in re-growth is found, this phenomenon which has been well documented with a variety of microorganisms treated with antimicrobial agents is known as Post Antibiotic Effect (PAE) [5-6]. It is reasonable to assume that delayed re-growth may depend upon the time that the cell needs to restore the physiological functions after non-lethal damage [7]. In particular, when the integrity of the envelope is compromised cytoplasmic material is lost leading to cell death, however, if the discharged molecules are not essential for the microorganism, its metabolic machine restores the lost compounds and resumes in various periods of time, depending on the magnitude of the damage, its physiological growth rate.

In this study Endox has been used to induce persistent growth suppression in representative of gram-positive and gram-negative bacteria as well as in *Candida parapsilosis*.

## MATERIALS AND METHODS

### Microorganisms

Bacterial strains used in this study included: *Enterococcus faecalis* (ATCC 29212), *Staphylococcus aureus* (ATCC 26927), *Pseudomonas aeruginosa *(ATCC 27853), *E. coli* (ATCC 25922) and *Candida parapsilosis* (ATCC 25019). *E. coli* K-12 C600 carrying a non conjugative plasmid pBP517 that contains the wild-type *gyr*A gene, and the determinant for amikacin-resistance, and a rifampin and nalidixic acid resistant C600 derivative strain, were used as donor and as recipient respectively, in testing the exchange of genetic material by HFAC. These latter strains were a generous gift of B.Wiedemann [8-9].

### Antimicrobial Agents

Sterile stock solutions of the drugs employed were prepared according to the instructions of the manufacturer by dissolving the compounds in the specific solvent to obtain a final concentration of 1 mg/ml.

### Susceptibility Tests

The minimum inhibitory concentrations (MICs) were determined in cation-supplemented Mueller-Hinton (CSMH) broth adopting the microdilution method following the procedure suggested by the Clinical and Laboratory Standards Institute [10].

### Preparation of the Microbial Suspension to be Exposed to the HFAC

Exponentially growing bacteria were washed by centrifugation and dispersed in water and glycerol (10%). This preparation was divided in aliquots and stored at -20°C until they were used for the experiments. After thawing, microbial cells were re-suspended in NaCl solution (0.1M) for small volume experiments and high molarity (1 M) for experiments carried out in greater volume. These two different experimental conditions were adopted after preliminary tests in order to gain reproducible results. Microrganisms were then exposed to HFAC generated by Endox. The number of microrganisms was determined by serial dilution of the original culture. Small aliquots (0.1 ml) of the cell suspension were then transferred, from a test tube containing the appropriate diluted sample, and streaked over the surface of an agar rich medium. When a low number of microbial cells was estimated, aliquots of 1 ml were mixed to soft agar (1.2 %) and poured on the agar plate. This last method was found to reduce many errors in the CFU/ml evaluation. After incubation at 36.5°C for 18-24 hours the number of viable cells was determined.

### Experimental Model

Endox was designed to delivery a 140ms pulse at a frequency of 312.5 KHz, and 1200KV/m. Under these conditions the number of survivors was reduced by more than 3 log and recombinants were not detected on selective plates. The time-pulse was then set at 60ms at a frequency of 300 KHz, and 800KV/m, this configuration was found to give reproducible results in genetical experiments (a 2-log reduction of viable bacteria). A structure was created that allows to expose the microorganisms *in vitro*. Such characteristic was obtained in a 200 μl pipette tip. In Fig. (**1**) is displayed the model used. Inside of the test tube the volume of the microbial suspension was 20 μl. At the level of the meniscus of the liquid the tip has been pierced with a needle connected to the neutral electrode of Endox. At the apex of the test tube, the active pole, which consists of a surgical steel needle, is inserted. The depth of this immersion modulated the different states of impedance. Three electromagnetic discharges were consecutively generated by the equipment, and then the number of survivors was determined by the CFU/ml dilution method.

### Exchange of Genetic Material Between Two E. coli Strains after Exposure to HFAC

A suspension of* E. coli* K-12 strains C600 carrying the non conjugative plasmid pBP517 (*gyr*A^+^) as donor was mixed with C600 rifampin (rif) and nalidixic acid (nal) resistant as recipient to achieve at least 5x10^8^ cells/ml in water glycerol. 0.2ml of the mixture was exposed to HFAC as above. 0.2 ml of the same suspension was not treated and used as control. Under these experimental conditions the number of viable bacteria was reduced by about 2 Log. Exposed and control samples were then added to 1.8 ml of SOC medium and incubated for 2 hours at 37°C. After incubation the samples were plated on selective plates and recombinants were scored after 48 hours at 37 °C.

### Spontaneous and Induced Plasmid Elimination from their Bacterial Hosts

Plasmid stability was evaluated as previously described in detail [11]. Briefly, bacterial strains carrying the plasmids with an initial inoculum of ≤10^3^ CFU/ml were grown for 20-24 generations (16-24 hours at 37° C), in drug-free medium and then diluted and plated on rich medium. After incubation for 18-20 hours at 37° C the colonies were replicated onto antibiotic-containing medium and medium containing no antibiotic. Growth on the appropriate drug-containing medium (amikacin) indicated the presence of the plasmid. In order to increase the number of plasmid-free bacteria the same experiment was carried out growing the microorganism in the presence of sub-inhibitory concentration of cefixime [12].

### Determination of Permeability Variation in Organisms Exposed to HFAC

A suspension of the microbial population to be analysed was adjusted to 10^6-8^ CFU/ml in water-glycerol (10%) depending on the organism studied. This sample was then divided in four parts, two containing the organism alone and the others were mixed with the antibiotic. The controls of the tests were two tubes with and without antibiotic. The other two samples were exposed to HFAC. The number of survivors was then evaluated by the CFU/ml dilution method.

### Determination of the Delay of Regrowth in Organism Exposed to HFAC

0,1 ml of the microbial suspension was adjusted to about 10^7^ CFU/ml in water-glycerol (10%), introduced into the tip and exposed to HFAC as above. The treated sample was then inoculated into 10 ml medium broth and incubated at 37° C for 6-7 hours. A similar volume of the same initial suspension was used as control. Viable count was carried out at time 0, and periodically each hour. The delay of regrowth was registered as the difference in time required by treated and non treated cultures of the same microorganism to increase by 1 log in CFU number.

## RESULTS

### Exchange of Genetic Material Between Two E. coli Strains after Exposure to HAFC

In order to verify the transfer of a non conjugative plasmid directly from strain to strain after exposure to HFAC, the first selection was carried out using plates containing amikacin (40 mg/L) and rifampin (100 mg/L). As reported in Table **1** in three different experiments a certain number of recombinants were found. All colonies were then tested for nalidixic acid resistance, the marker of the recipient strain. All the recombinants were susceptible to this antibiotic indicating the presence of the plasmid which, in addition to the amikacin resistance marker, includes the wild-type *gyrA* gene. In the diploid strain, in fact, susceptibility is dominant over resistance. Two different recipient strains, randomly selected were then used as donor in further experiments. Using a spontaneous sodium azide-resistant derivative of *E. coli* ATCC25922 as recipient organism, the new mating mixture was exposed to HFAC and recombinants were again found (Table **1**). These new recipients obtained were again used as donor employing as recipient strains the rifampin and nalidixic acid resistant organism of the first mating experiments (C600 nal, rif). The recombinants found showed amikacin resistant phenotype together with nalidixic acid susceptibility. The presence of the plasmids in the recipient bacteria was also confirmed by observing the recovery of the original phenotype when strains were cured of plasmids by long term sub-culture on non selective medium or by sub-culture in the presence of sub-MIC cefixime to promote plasmid loss (data not shown).

### Determination of Permeability Variation in Organisms Exposed to HAFC

**Fig. (2):**
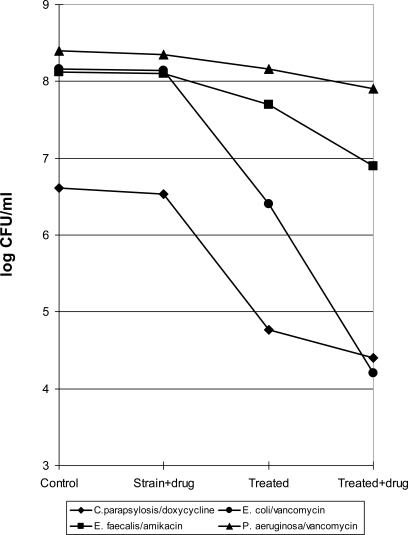
Survivors of strains tested exposed to HFAC alone or in the presence of antibiotic

Membrane permeability was tested with organism-antimicrobial combination where the drug selected is not normally active on the microbe but likely to be active if the permeability barrier is lessened and the drug gets inside the cell (Fig. **2**). When *Ps. aeruginosa* was combined with vancomycin, as expected, there was no significant variation in the number of CFU/ml found in comparison with the original inoculum. The application of an electromagnetic field to the culture produced a low decrease in the number of viable cells and the combination of HAFC with vancomycin resulted bactericidal for more than 50% of the initial bacterial population. In considering *Ent. faecalis* in combination with amikacin, no lethal activity was demonstrated by this antimicrobial against this strain, however the exposure to electric field reduced the number of CFU/ml detected and the association of HFAC with amikacin was markedly bactericidal on about 90% of the initial bacterial population.

The combination of the electric field and vancomycin was marked bactericidal against *E. coli*. In fact the number of viable cell found after this exposure was reduced by 4 Log in combination with the original concentration. This species resulted also susceptible to the treatment of the HFAC alone with the reduction of more than 95% of the total cells exposed while the antibiotic alone as expected was not active against this strain.

*Candida parapsilosis* resulted susceptible to the electric field with a significant reduction of about 2 log of the microorganism treated, in addition of doxycycline increased the bactericidal effect by about 50%.

### Determination of the Delay of Regrowth in Organisms Exposed to HFAC

**Fig. (3):**
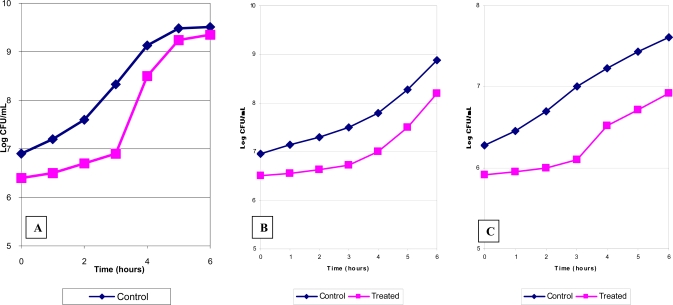
Persistent growth suppression induced by HFAC in *Ent.faecalis* (**A**) *Ps.aeruginosa* (**B**) and *Candida parapsilosis* (**C**).

The delay of regrowth after HFAC treatment was studied in representative organisms of gram-positive, gram-negative bacteria and yeast. Following HFAC survivors of *Ent. faecalis* (Fig. **3A**), *Ps. aeruginosa* (Fig. **3B**) , and *C. parapsilosis* resumed their original growth rate after delay 0f 1.0, 0.75, and 0.6 h respectively compared their appropriate controls.

## DISCUSSION

Present findings indicate that the HFAC generated by Endox allows the transfer of a non conjugative plasmid between two different *E. coli* strains, promotes the entry into the bacterial cell of antibiotics that are not normally able to interact with the microorganism because of a natural permeability barrier, and induces a delay in the re-growth of survivors of different species following treatment.

The exchange of genetic material among *E. coli* cells and the uptake of selected antibiotics by the exposed organisms is reminiscent of the electroporation method for delivery of foreign molecules into bacterial cells. This probably reflects the mode of action of Endox, which by generation of high frequency alternating current may create pores in the envelope of the microrganisms allowing ingress and egress of key molecules and may duplicate many of the applications described for the standard electroporation techniques [4]. In the present study the exchange of the genetic material was detected between two *E. coli* strains. HFAC generated by Endox may represent a quicker, cheaper and more convenient alternative to conventional electroporation systems.

The uptake of different antibiotics was another important observation of this study. This might suggest the use of various compounds in combination with HFAC to increase the lethality of the treated organisms. This should be the case of endodontic treatment, the use for which Endox was originally developed [1] and for topic therapy to enhance the beneficial effect of both the antimicrobial and the HFAC.

Finally a period of re-growth suppression was found with all the microorganisms tested. This is another interesting point observed in the experimentation suggesting that after HFAC exposure, especially during therapy, the antimicrobial effect lasts for a long time. This phenomenon is similar to that observed in the postantibiotic effect [6]. Since this characteristic is considered an additive property for the antimicrobials [13-14] Endox appears to be endowed of all the characteristics of an antimicrobial agent without selecting resistant microorganisms.

Taken together the present findings indicate that Endox is a versatile instrument that when is used for endodontic treatment causes the death of the microorganism exposed by promoting damages in the envelope of the cell. On the other hand survivors required a long period of time to recover the normal growth rate that improve the beneficial effects of this treatment. The possibility of a direct transfer of genetic material from strain to strain should be taken into account when genetical experiments are planned with more specific instruments that required expensive waste of time and materials

## Figures and Tables

**Fig. (1):**
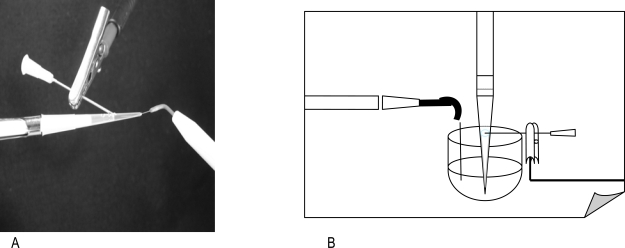
Experimental model reproducing the environment where microorganisms have been exposed to HFAC. **A**, Test tube containing 20 µl of bacterial suspension punctured at the liquid top, to the apex a probe of Endox is applied. **B**, the probe of Endox is emerged in liquid outside the test tube

**Table 1.** Effect of HFAC on Non-Conjugative Plasmid Transfer in *E. coli*

Donor	Recipient	Experiment	N. recombinants/ 10^7^recipients^*^
C600 (pBP517, gyrA+)	C600 nal, rif	Ia	15
		IIa	12
		IIIa	21
		control	0
C600 (pBP517) rif	ATCC25922 azi	Ib	7
		IIb	14
		control	0
ATCC25922 (pBP517) azi	C600 nal, rif	Ic	18
		IIc	27
		control	0

Nal, nalidixic acid-resistant (100 mg/L); rif, rifampin-resistant (100 mg/L) az, spontaneous sodium azide-resistant (200 mg/L) strain ^*^The number was determined after exposure to HFAC
